# Evaluation of myocardial injury patterns and ST changes among critical and non-critical patients with coronavirus-19 disease

**DOI:** 10.1038/s41598-021-84467-4

**Published:** 2021-03-01

**Authors:** Anam Liaqat, Rao Saad Ali-Khan, Muhammad Asad, Zakia Rafique

**Affiliations:** 1 Riphah International University, Islamabad, Pakistan; 2 Pak Emirates Military Hospital, Rawalpindi, Pakistan; 3 Armed Forces Institute of Cardiology, Rawalpindi, Pakistan

**Keywords:** Cardiology, Diseases

## Abstract

Novel coronavirus disease (COVID-19) has led to a major public health crisis globally. Currently, myocardial damage is speculated to be associated with COVID-19, which can be seen as one of the main causes of death of patients with COVID-19. We therefore, aim to investigate the effects of COVID-19 disease on myocardial injury in hospitalized patients who have been tested positive for COVID-19 pneumonia in this study. A prospective study was conducted among 201 patients with COVID-19 in the Pakistan Military Hospital from April 1 to August 31, 2020, including non-critical cases and critical cases. COVID-19 patients were stratified as critical and non-critical according to the signs and symptoms severity; with those requiring intensive care and invasive mechanical ventilation as critical, and those did not requiring invasive mechanical ventilation as non-critical. A total of 201 COVID-19 patients with critical and non-critical categories presented with myocardial injury. All patients with myocardial injury had an elevation in CKMB and Troponin-I levels. Of these patients, 43.7% presented with new electrocardiography (ECG) changes, and ST depression was typically observed in 36.3% patients. In addition, 18.7% patients presented with abnormal echocardiography findings, with right ventricular dilatation and dysfunction commonly seen among critical group patients. Results analyzed by a logistic regression model showing COVID-19 direct contribution to myocardial injury in these patients. COVID-19 disease directly leads to cardiovascular damage among critical and non-critical patients. Myocardial injury is associated not only with abnormal ECG changes but also with myocardial dysfunction on echocardiography and more commonly observed among critical patients.

## Introduction

Coronavirus disease 2019 (COVID-19) has emerged in the world as both a pandemic and a major public health crisis in China, as well as for the international community. Globally, as of there have been 49,578,590 confirmed cases of COVID-19, including 1,245,717 deaths, reported to WHO recently^1^. Serious acute respiratory syndrome coronavirus 2 (SARS-CoV-2) can be spread from person to person via respiratory droplets and direct contact, creating a major public health challenge^2^. In addition to concentrating on severe pneumonia in patients, the SARS-CoV-2 can invade several essential organs and cause multiple organ failure with cytokine storms. The heart is one of the most critical organs and it is particularly probable that SARS-COV-2 infection also contributes to viral myocardial damage, which could also be considered one of the leading causes of COVID-19 patients' death^3^.

Previous studies have shown the noval insight into the occurrence and implications of SARS-COV-2 induced myocardial injury. Shi et al.^4^ present a retrospective study of 416 COVID-19 hospitalized patients, 19.7% of whom had evidence of myocardial injury evidenced by elevation of troponin-I (TnI) high-sensitivity levels, and higher levels of TnI were associated with higher mortality rates among those with myocardial injury. Guo et al.^5^ recorded related findings in 187 COVID-19 diagnosed patients, of which 27.8% had myocardial damage as calculated by high levels of troponin-T (TnT), and the highest mortality rates were found in those with high levels of TnT that had cardiovascular disease, i.e. 69.4%. Nevertheless, mortality rates of people with elevated levels of TnT without previous cardiovascular disease were also significant and was 37.5%. Further they showed the important correlation of TnT levels with levels of C-reactive protein and N-terminal pro-B-type natriuretic peptide (NT-proBNP), thereby connecting myocardial damage to the inflammatory extent and ventricular dysfunction. Yang and Zin^6^ follow up on these lines of evidence. They observed that patients having pre-existing cardiovascular conditions are more prone to adverse effects of COVID-19 disease. Notably, Shi et al. Guo et al. and Yang and Zin's papers from China also discussed the unique marked sensitivity of SARS-CoV-2 to the host angiotensin-converting enzyme 2 (ACE-2) receptor, increasing the likelihood for direct viral endothelium and myocardium infection and myocarditis.

At present, there is insufficient evidence available to resolve any specific threats of COVID-19 induced myocardial injury. This awareness is of paramount importance to understand the risk for direct and indirect adverse effects of the SARS-CoV-2 on the heart, particularly in those with already existing heart disease. In this study, we aimed to investigate the myocardial injury patterns, ECG changes, echocardiographic parameters in COVID-19 diagnosed patients of critical and non-critical categories in Pakistan’s largest tertiary care center. These results will provide updated information for physicians to improve the survival of people with COVID-19.

## Methods

### Study design and population

This prospective study is an observational single-center which was conducted at the Military Hospital, Rawalpindi, Pakistan, one of the largest tertiary teaching hospitals, declared as the official center for COVID-19 patients. We included people who had COVID-19 hospitalizations from April 2020 till August 2020. We have followed the patients who were admitted to the hospital due to Covid-19 disease on the basis of presentation of signs and symptoms severity (i.e. non-critical and critical ones). Not all Covid-19 patients presented to the hospital were enrolled in the study. Patients who did not require admission to the hospital were excluded from the study. Clinical data was collected by the attending physician during hospitalization of patients within 48 h.

This study complies with the edicts of the 1975 Declaration of Helsinki^7^, and approval was taken by the institutional ethics board of the Military Hospital of Pakistan Reference number A/28/BC/215/2020. Informed consent was taken from the attendants and stable patients.

### Diagnosis and grading of COVID-19

All patients were diagnosed and graded as per the World Health Organization's interim guidance^8^. The diagnosis of COVID-19 was confirmed with real-time reverse transcriptase-polymerase chain reaction (RT-PCR). A chest computed tomographic (CT) scan was performed in all positive patients to see pathological changes in the lungs. COVID-19 patients were divided into two groups as non-critical and critical patients. Critical patients were included: (1) Patients with one of the three conditions; respiratory failure (involves mechanical ventilation), shock, and organ failure (requires intensive care)^9^: (2) suffering from high respiratory frequency (RR ≥ 30 bpm), oxygen saturation ≤ 93%, and PaO_2_/FiO_2_ ratio ≤ 300 mmHg. Non-critical patients included those with oxygen saturation of 94% or less and RR equals to or less than 25 breaths per minute, and did not requiring invasive mechanical ventilation during admission.

The clinical data of patients were collected from electronic medical records, including demographics, clinical symptoms, and signs, co-existing conditions, imaging findings, laboratory results.

### Myocardial injury patterns

Myocardial injury was defined as a rise and fall in cardiac TnI with at least one value above the 99th percentile upper reference limit (URL), attributable to cardiovascular and non-cardiovascular causes^10^, and myocarditis related abnormalities defined as a triple elevation in cardiac TnI (over 0.12 ng/mL) plus abnormalities on echocardiography and/or ECG according to American Heart Association (AHA)^11^.

In all patients, ECG was done at the time of admission. ECG was recorded with standards of 25 mm/s and 1 mV/cm calibration, and 0.05–150 Hz filter setting. ECG tracings were coded and analyzed off-line. Minnesota Coding system was used to label the ECG changes^12^. Any arrhythmia occurring during the hospital stay was also assessed.

Transthoracic echocardiography (TTE) was done in all patients, by cardiologist having experience in performing echocardiograms, within 24 h of being hospitalized. All examinations were performed at bedside in covid dediated units by taking proper safety measures. Echocardiographic images were taken by following American Society of Echocardiography (ASE) guidelines^13^. Left ventricular ejection fraction (LVEF) was assessed by using Simpson’s biplane method in apical 4- and 2-chamber views and by parasternal long-axis view to measure the left ventricular end diastolic diameter (LVEDD), the left ventricular end systolic diameter (LVESD) to calculate fractional shortening. Right ventricular (RV) diameter was measured from the apical 4-chamber view at the basal third of the right ventricle and at midcavity in end-diastole. RV systolic function was assessed by measuring TAPSE in apical four chamber view in M Mode by placimg cursor at lateral tricuspid valve annulus. For every echocardiography exam proper disinfection guidelines were followed and echocardiography machine specified for covid unit only was used.

### Statistical analysis

Categorical variables are presented as percentages, and continuous variables as mean and standard deviation (SD). Categorical variables are gender, symptoms such as chest pain, SOB, comorbidities (Cardiovascular disease, Chronic Lung disease, smoking, obesity, Hypertension, diabetes) ECG pattern, echocardiographic findings, during hospitalization. The Chi-Squared exact test for categorical variable and independent t-test for continuous variables were used. The differences in the means for TnI, d-dimer, C-reactive protein CRP, creatinine phosphokinase CPK, creatinine kinase-MB CKMB, Serum Urea, age temperature, respiratory rate, body mass index BMI, number of co-morbidities were analyzed using independent t-test. Multivariate logistic regression analysis was used to assess the association between severity status (critical and non-critical) and cardiac markers (TnI or CKMB). All statistical analyses will be performed with SPSS, version 24.0 with 0.05 as a level of significance.

## Results

### Demographic and clinical characteristics

There were 201 hospitalized patients with COVID-19 disease (Table 1), including 119 male (59.2%) and 82 (40.8%) female. The mean age was 44.62 ± 15.2, and the mean BMI was 24.73 ± 2.77. There were 144 (71.6%) patients presented as non-critical and 57(28.3%) as critical categories. Overall more males than females were affected and males constituted the majority of the critical patients' group. The most common symptoms were fever [100.3 ± 1.1], chest pain/tightness [22 (38.6%)], and shortness of breath [57 (100%)] identified among patients of the critical group as compared to patients in the non-critical group. However, respiratory rate and systolic blood pressure were higher in both critical and non-critical patients with mean and SD values of [26.5 ± 1.3/21.7 ± 2.3] and [138.6 ± 7.8/125.2 ± 9] respectively. P-value was < 0.001. Comorbidities data shown in Table 1 represents that out of 201 total number of enrolled patients, critical patients are presented with more comorbidities as compared to non-critical patients with p < 0.001, and most common co-morbidities were diabetes (16.4%), hypertension (14.4%), and cardiovascular disease (13.4%) among them.

**Table 1:** Demographics and clinical characteristics of Covid-19 patients.

Characteristics	Total	Non-critical	Critical	*P*-value
No. of patients	201	144	57	
Age (years)	44.6 ± 15.2	39.1 ± 12.7	59.4 ± 10.9	< 0.001
Male	119 (59.2)	85 (58.2)	34 (61.8)	0.643
Female	82 (40.8)	61 (41.8)	21 (38.2)	
Shortness of breath	77 (38.3)	20 (13.9)	57 (100.0)	< 0.001
Chest pain	23 (11.4)	1 (0.7)	22 (38.6)	< 0.001
BMI (kg/m^2^)	24.7 ± 2.8	23.8 ± 2.1	27.3 ± 2.7	< 0.001
Temperature (F)	99.0 ± 1.1	98.5 ± 0.7	100.3 ± 1.1	< 0.001
Respiratory rate (per min)	23.0 ± 2.9	21.7 ± 2.3	26.5 ± 1.3	< 0.001
Diastolic BP (mmHg)	79.8 ± 7.9	77.5 ± 6.4	86.2 ± 7.9	< 0.001
Systolic blood pressure (mmHg)	128.9 ± 10.5	125.2 ± 9.0	138.6 ± 7.8	< 0.001
Blood saturation of oxygen (%)	96.9 ± 2.3	97.4 ± 1.8	95.7 ± 2.9	< 0.001
**Comorbidities**				
Hypertension	35 (17.5%)	6 (3%)	29 (14.4%)	< 0.001
Diabetes	35 (17.5%)	2 (1%)	33 (16.4%)	< 0.001
Cardiovascular disease	30 (14.9%)	3 (1.5%)	27 (13.4%)	< 0.001
Chronic lung disease	5 (2.5%)	0 (0.0%)	5 (2.5%)	0.002
Smoking	33 (16.4%)	13 (6.5%)	20 (10%)	< 0.001
Obesity	10 (5%)	0 (0.0%)	10 (10%)	< 0.001
Cancer	8 (4%)	4 (2.0%)	4 (2%)	0.242

### Evaluation of cardiac markers

TnI were raised over (0.04 ng/ml) [Mean ± SD 0.07 ± 0.12] among non-critical patients and triple elevated over (0.12 ng/ml) among critical group patients during hospitalization (p < 0.001). LDH, d-Dimers, Ferritin were also raised among the critical group compared to the non-critical group, showing poor prognosis in these patients. Patients in both groups showed elevated CRP with p < 0.001. Critical patients had significantly low WBC counts and platelet counts (p value < 0.001). ALT and ALP also showed an increased mean value among critical patients. Lymphocyte counts were lower in the critical group with p value < 0.001 (Table 2).

**Table 2:** Comparison of specific and non-specific cardiac injury markers and laboratory parameters between critical and non-critical cases.

Laboratory parameters	Non-critical group	Critical group	P-value
Troponin-I (< 0.04 ng/mL) Poor prognosis > 0.12 ng/mL	0.07 ± 0.12	0.48 ± 0.32	< 0.001
d-Dimer (< 500 ng/mL) Poor-prognosis > 1000 ng/mL	304.87 ± 164.55	1089.35 ± 409.21	< 0.001
CRP (< 8 mg/L)	20.81 ± 28.63	153.15 ± 72.29	< 0.001
LDH (U/L)	169.55 ± 100.88	528.65 ± 211.81	< 0.001
Ferritin (µg/L)	197.14 ± 245.45	1417.15 ± 525.97	< 0.001
CPK (< 192 U/L)	163.28 ± 20.02	213.15 ± 17.87	< 0.001
CK-MB (< 25U/L)	23.04 ± 8.83	45.56 ± 12.17	< 0.001
WBC (×10^3^/µL)	6.58 ± 1.38	5.26 ± 2.26	< 0.001
Absolute lymphocyte count (µL)	3717.0 ± 1163.69	1484.95 ± 836.95	< 0.001
Platelets (×10^3^/µL)	253.22 ± 71.36	127.63 ± 33.33	< 0.001
Serum urea (mmol/L)	5.30 ± 0.95	4.82 ± 1.17	0.003
Serum-creatinine (µmol/L)	89.03 ± 13.22	123.55 ± 15.34	< 0.001
Serum sodium (mEq/L)	138.93 ± 8.81	135.20 ± 2.14	< 0.001
Serum potassium (mEq/L)	4.5 ± 2.85	4.5 ± 0.28	0.837
Serum bilirubin (µmol/L)	18.18 ± 4.23	31.82 ± 16.10	< 0.001
ALT (< 42 IU/L)	44.01 ± 20.92	98.87 ± 45.84	< 0.001
Alkaline phosphatase (< 150 IU/L)	111.38 ± 27.07	143.67 ± 25.10	< 0.001

### ECG findings

From 144 non-critical patients and 57 critical patients enrolled in the study, abnormal ECG changes were seen among 33/144 (22.9%) non-critical VS 55/57 (96.5%) critical subjects, total 88/201 (43.7%) patients, while ECG findings of 113/201 (56.21%) patients were normal [111/144 (77.0%) non-critical VS 2/57 (3.5%) critical patients]. Among 88 patients presented with abnormal ECGs, sinus tachycardia was present in total 59/88 (67.04%) patients [16/59 (27.1%) non critical VS 43/59 (72.8%) critical]. Atrial fibrillation was the only arrhythmia occurred in total 21/88 (23.8%) patients [4/21 (19.0%) non-critical VS 17/21 (80.09%) critical] with abnormal ECGs. In addition, ST-T changes were typical ECG abnormalities found in COVID-19 disease patients. Anteroseptal ST depression was seen in total 32/88 (36.6%) patients [12/33 (36.6%) non critical VS 20/55 (36.36%) critical], followed by Anteroseptal T wave inversions in 21/88 (23.86%) patients [9/33 (27.2%) non-critical VS 12/55 (21.8%) critical category], and Anteroseptal ST elevation in 9/88 (10.22%) patients [2/33 (6.06%) non-critical VS 7/55 (12.7%) critical] respectively. Another common ECG abnormality seen was right bundle branch block (RBBB) observed in total 23/88 (26.1%) patients, with 7/33 (21.2%) comprises non-critical and 16/55 (29%) critical categories respectively. Overall, critical patients were found to have more abnormal ECG findings as compared to non-critical ones, and reached statistical significance in critical covid-19 disease patients with p < 0.001.

### Echocardiography findings

Echocardiography was performed in 181 out of 201 patients enrolled for study. 147/181 (81.2%) patients displayed normal Echo findings. Echo findings suggest that 25/181 (13.8%) patients showed RV dilatation and RV dysfunction, 5/181 (2.76%) patients with LV dysfunction only (1 non-critical patient and 4 critical patients), and 4/181 (2.2%) patients reported with biventricular dysfunction of critical category. None of the non-critical group patients showed any abnormal Echo changes except in one patient with LV dysfunction.. Among non-critical and critical patients 1/181 (0.5%) VS 3/181 (1.65%) patients showed EF less than 40%, 15/181 (8.2%) VS 32/181 (17.6%) with EF between 40–59%, and 112/181 (61.8%) VS 18/181 (9.9%) with EF > 60% respectively (Table 3).

**Table 3:** Echo and ECG assessment by critical and non-critical cases.

Items	Non-critical group	Critical group	p-value
Normal ECG	111 (77.0%)	2 (3.5%)	< 0.001
**Abnormal ECG**	33 (22.9%)	55 (96.5%)	
Tachycardia	16 (27.1%)	43 (72.8%)	< 0.001
Atrial fibrillation	4 (19%)	17 (80.09%)	< 0.001
Anteroseptal ST depression	12 (36.6%)	20 (36.6%)	< 0.001
Anteroseptal T wave inversion	9 (27.2%)	12 (21.8%)	
Anteroseptal ST elevation	2 (6.06%)	7 (12.7%)	
Q wave inferior leads	3 (9.09%)	0 (0.0%)	
RBBB	7 (21.2%)	16 (29.0%)	
Normal echo	126 (69.6%)	21 (11.6%)	< 0.001
**Abnormal echo**	1 (0.5%)	33 (18.23%)	
LV dysfunction	1 (0.5%)	4 (2.2%)	< 0.001
RV dilatation, RV dysfunction	0 (0.0%)	25 (13.8%)	
Biventricular dysfunction	0 (0.0%)	4 (2.2%)	
**Ejection fraction**			
≥ 60%	112 (6.8%)	18 (9.9%)	< 0.001
40–59%	15 (8.2%)	32 (17.6%)	
< 40%	1 (0.5%)	3 (1.65%)	
Discharge patients	144 (77.0%)	43 (22.9%)	< 0.001
Death	0 (0.0%)	14 (6.96%)	

### Cardiac markers and risk of severity and mortality

Table 4 presents the association between severity of COVID and cardiac enzymes/abnormal Echo or ECG using logistic regression. We found a significant association between severity (critical or non-critical) and cardiac markers. People with ciritical symptoms are significantly higher odds of TnI than people without critical symptoms. The effect sizes were consistently large for the outcomes of CKMB, abnormal Echo and ECG, compared to people without critical symptoms. There were 14 deaths reported in this study. All of them were critical cases with abnormal Echo and ECG findings, and less than 60% ejection fraction.

**Table 4:** Adjusted odds ratios (95% CI) of cardiac injury markers, by severity status (*n* = 201).

Items	OR (95% CI)		p-value
	Non-critical group	Critical group	
Elevated troponin-I (> 0.04 ng/ml)	n/N = 68/144	n/N = 55/57	
	1.0	4.10 (0.69–24.39)	0.120
Elevated troponin-I (> 0.12 ng/ml)	n/N = 11/144	n/N = 45/57	
	1.0	8.14 (2.13–31.10)	0.002
Elevated CKMB (≥ 25U/l)	n/N = 50/144	n/N = 56/57	
	1.0	10.56 (1.15–96.63)	0.037
Abnormal echo	n/N = 1/127	n/N = 33/54	
	1.0	5.21 (0.47–57.84)	0.179
Abnormal ECG	n/N = 33/144	n/N = 55/57	
	1.0	28.22 (4.04–196.81)	< 0.001

ORs were adjusted for age, gender, BMI, and comorbidity. Logistic regression. Non-critical was referent.

## Discussion

Coronaviruses belong to the ribonucleic acid (RNA) viruses’ family named Coronaviridae, present in both humans and mammals. It is an enveloped virus that can cause respiratory, enteric, hepatic, and neurological disease. Although it has been linked to diseases involving the pulmonary tract, there is also an association with cardiac pathologies^14^. Special attention was paid to the function of angiotensin-converting enzyme 2 (ACE2), a protein that is proposed to be the binding receptor for SARS-CoV-2 and allows its cellular entrance. ACE2 is found in epithelial cells of lung alveoli, and also highly expressed in adult human heart pericytes, suggesting an inherent heart vulnerability to SARS-CoV-2 infection. Despite ACE2 mediated entry, SARS-CoV-2 also down-regulates the expression of ACE2 receptors, resulting in a diminished conversion of angiotensin II (Ang-II) to cardioprotective angiotensin 1–7 protein. Besides the heart and lungs, Ang-II conversion to 1–7 in vascular endothelium, intestinal epithelium, and the kidneys leads to the inhibition of vasoconstrictor, pro-inflammatory, pro-oxidant, pro-proliferative, and pro-fibrotic functions mediated by Ang-II through AT1 receptors^15^. Thus, suppression of ACE2 expression and subsequent rise in Ang-II levels in COVID-19 patients may pose a further danger to both the heart and vessels.

Although mainly research has been done on pulmonary complications, few investigations have been done to evaluate the cardiovascular effects associated with Covid-19. Elevated cardiac TnI has been observed since the first data analyses in China, representing myocardial damage as a potential pathogenic pathway that contributes to disease severity and mortality among COVID-19 patients. The phenomenon of elevated cTn levels in COVID-19 patients may be explained by several mechanisms such as viral myocarditis, myocardial damage driven by cytokine syndrome, microangiopathy, and unmasked coronary artery disease. It has been proposed that microvascular injury in patients with COVID-19 causes perfusion disorders, hyper-permeability of the vessels, and vasospasm, resulting in myocardial injury^16^.

Research conducted by Wang et al. demonstrated the association between clinical comorbidities and disease severity among COVID-19 disease patients^17^. In our study, there was a significant association between clinical comorbidities and disease severity including diabetes, hypertension, obesity, and smoking. However, cancer patients did not show any significance. Although Robilotti et al.^18^ showed that there was a substantial rate (20%) of severe respiratory outcomes among cancer patients, there study results were contradictory to our findings. Evidence suggests that the development of cancer is linked with a blunted immune status depicted by an increase in immunosuppressive cytokines along with suppressed induction of pro-inflammatory danger signals, impairment in the maturation of dendritic cell, and role of immunosuppressive leukocytes^19^.

Studies have shown that elevated TnI levels may be seen in 7–17% of patients hospitalized with COVID-19 and 22–31% of those admitted to the intensive care unit^9^. In this study, out of 201 patients, 54% had raised TnI levels suggesting myocardial injury, and 47.2% patients lie in the critical group. TnI levels were significantly higher > 0.2 ng/ml in critical patients, even though mean values were raised in both groups. Increased levels of cTn were also associated with abnormal ECG findings. Other studies have also shown that heart injury and elevated TnI may contribute to complications including ventricular fibrillation, acute coagulopathy, electrolyte disturbances, acute kidney failure, and the necessity for mechanical ventilation^5^. Furthermore, the autopsy report of people who died due to the 2002 SARS outbreak revealed that 35% of heart specimens demonstrated the existence of viral RNA in the myocardium, and this, in turn, was linked to lower expression of ACE2 protein^20^, SARS-CoV-2 can represent the same mechanism because the two viruses seem to be similar in the genome^21^. Also, patients with pre-existing or with risk factors for coronary artery disease have a high chance of developing severe coronary syndrome following acute infections, as shown in epidemiological and clinical influenza studies and other acute inflammatory conditions. This may arise due to an imbalance between myocardial oxygen supply and demand, such that the TnI elevation may be viewed as a type 2 myocardial infarction (MI). Decreased oxygen supply in COVID-19 patients is usually triggered by hypoxic respiratory failure and is a sign of disease severity. Infectious conditions, by comparison, are frequently followed by fever, tachycardia, and endocrine dysregulation, contributing to a marked rise in demand for myocardial oxygen. Besides, hypoxemia also results in elevated intracellular calcium with subsequent apoptosis of the cardiac myocytes^22^.

Circulating cytokines produced during extreme systemic inflammatory stress may result in instability and breakup of atherosclerotic plaque with thrombus formation contributing to type 1 MI due to COVID-19^23^. Furthermore, due to the expression of ACE2 in endothelial vascular cells, a direct viral vascular infection that contributes to plaque instability also contributes to type1 MI in patients with COVID-19^24^.

Coagulopathies seen in severe COVID 19 cases can be explained by endothelial dysfunction, cytokine storm, oxidative stress, and activation of Ang II. A postmortem report from Singapore states that 4 out of 8 patients infected with SARS had pulmonary thrombo-embolic lesions and 3 had deep vein thrombosis. To date, only 1 case of pulmonary embolism associated with COVID-1939 has been reported, but only half of the COVID-19 patients have raised levels of D Dimer which is associated with more deaths^25, 26^. Increased d-dimer levels can be explained by inflammatory reactions, which stimulate severe fibrinolysis in the lungs with overspill into the bloodstream ^27^. In our study, analysis of laboratory results revealed that d-dimers and inflammatory markers were significantly raised in critical patients. Also, critical patients presented with significantly low levels of WBC count, platelet counts, Lymphocyte counts. ALT and AST also showed increased mean values among critical patients.

Although, the incidence of the acute coronary syndrome and MI in infected patients during the first SARS outbreak was described. However, in COVID-19, very limited details are available on the changes in electrocardiogram linked to MI. Angeli et al.^28^ showed from their study on 50 patients that ECG changes were present in patients with COVID-19 disease irrespective of the severity of the disease. Common ECG changes were seen in 43.7% of patients in our study, Sinus tachycardia was the common followed by Anteroseptal ST Depressions irrespective of disease severity. ECG changes also when correlated with TnI level suggested that their values were higher as compared to ones having normal ECG (p-value < 0.001). Also, ECG changes had a significant relation with the mortality as well (p-value < 0.001) among COVID-19 disease patients which are positively related to the severity of the disease.

Due to multidimensional involvement of the cardiovascular system by covid 19 disease role of echocardiography is emerging. It offers a quick guide of cardiac function noninvasively and can also help in evaluation of hemodynamic status of the patient^29^. Both left and right heart chambers have been considered to be involved in the disease process. Peng et al.^30^ described the abnormal echocardiography findings in covid pneumonia patients, including decrease in the systolic and/or diastolic function of the heart, takotsubo cardiomyopathy, RV enlargement, acute pulmonary hypertension, increased cardiac output, and higher LVEF (Hyperdynamic cardiac function). Another research conducted by Barman et al.^31^ showed that RV, RA, and inferior vena cava dilatation, increased systolic pulmonary artery pressure, decreased RV-fractional area change were typical echocardiographic findings in serious covid-19 diagnosed patients, whilst also LVEDD, LVESD, and LA diameters were greater, and LVEF, E wave, E/A ratios were lower. Hani et al.^32^ recorded RV dilatation in 41%, RV dysfunction in 27%, hyperdynamic or normal LV in 89% covid-19 disease patients in his study. A systematic analysis conducted by Sczekely et al.^33^ in 100 patients with Covid-19 infection found that the most prevalent echocardiographic trend was RV dilatation with or without dysfunction (39% patients), accompanied by LV diastolic dysfunction (16% patients), LV systolic dysfunction (10% patients), and valvular heart disease (3 patients) undergoing echocardiographic evaluation within 24 h of admission. There was a normal echocardiogram in the remaining 32 patients (32%). In our study 99.2% of patients of the non-critical group and 38.9% of patients of the critical group displayed normal echocardiography. Only 1 patient among the non-critical group presented with LV dysfunction in echocardiography as compared to 33(61.1%) patients of the critical category with RV dilatation and dysfunction being most common, (in 25 patients, possibly due to parenchymal or vascular disease/resistance) followed by biventricular dysfunction in 11.7% patients, and LV dysfunction in 7% of patients respectively, suggesting SARS-COV infection severity in cardiac damage especially among critical group patients. While the epidemic of COVID-19 infection began months ago, comprehensive echocardiographic examination of patients with COVID-19 has not been conducted regularly. An echocardiogram can be useful in recognizing the etiology of cardiac injury and potential targeted treatment especially among clinically deteriorating patients.

## Limitations

There are a few limitations to our study. This was a single-center experience with a relatively small sample size therefore, further studies with a large population size may help to draw a definitive conclusion regarding the severity of disease and ECG changes. Further, a detailed evaluation of echocardiographic changes was not studied owing to the short study period and the limited amount of time due to the disease’s peak period going on, so observing more parameters with better modalities might give a broader picture of cardiac involvement. In addition, the reversibility of the electrocardiographic changes with an improvement of the clinical status could not be discerned.

## Conclusions

COVID-19 disease is associated with multi-system involvement including the cardiovascular system. In conclusion, myocardial involvement presenting as myocardial injury is more commonly seen in critically ill covid patients in the form of elevated troponin levels and ECG abnormalities (including ST changes and arrhythmias), and there is a positive correlation between death and markedly elevated troponin levels and ECG abnormalities. While echocardiography showed a predominance of ventricular dysfunction in critical patients, there was no substantial association with mortality. As cardiac involvement in critical patients may be considered to alter the course of the disease, close examination of cardiac status should be taken into account.
